# Electrophysiological Predictors of Clinical Outcome in Traumatic Neuropathies: A Multicenter Prospective Study

**DOI:** 10.1155/2016/4619631

**Published:** 2016-07-28

**Authors:** Palma Ciaramitaro, Mauro Mondelli, Eugenia Rota, Bruno Battiston, Arman Sard, Italo Pontini, Giuliano Faccani, Giuseppe Migliaretti, Aristide Merola, Dario Cocito, Italian Network for Traumatic Neuropathies

**Affiliations:** ^1^EMG Service, CTO Hospital, AOU Città della Salute e della Scienza di Torino, Torino, Italy; ^2^EMG Service, Azienda Sanitaria Locale 7 Siena, Siena, Italy; ^3^Neurology Division, Piacenza Hospital, Piacenza, Italy; ^4^Traumatology Division, CTO Hospital, AOU Città della Salute e della Scienza di Torino, Torino, Italy; ^5^Hand Surgery Division, CTO Hospital, AOU Città della Salute e della Scienza di Torino, Torino, Italy; ^6^Neurosurgery Division, CTO Hospital, AOU Città della Salute e della Scienza di Torino, Torino, Italy; ^7^Public Health and Microbiology Department, University of Torino, Torino, Italy; ^8^Department of Neurosciences, Molinette Hospital, Università degli Studi di Torino, Torino, Italy; ^9^Associazione Polineuropatie Croniche Piemonte ONLUS, Torino, Italy; ^10^GdS Neuropatie Traumatiche e Iatrogene, SINC (Società Italiana di Neurofisiologia Clinica), Via Nizza 45, 00198 Roma, Italy

## Abstract

*Objectives*. This prospective, observational, multicentre study aims to identify electrodiagnostic (EDX) markers of clinical recovery in patients with traumatic neuropathy (TN) receiving surgical (S) and nonsurgical (NS) treatments.* Methods*. Subjects referred to the Italian Traumatic Neuropathy Network between 2010 and 2011 (307 patients, for a total of 444 TN) were evaluated with serial clinical/EDX evaluations at 6, 12, 24, and 36 months of follow-up.* Results*. Primary surgery was performed in 21 subjects with open lesions and evidence of neurotmesis, while closed lesions were treated with either conservative medical approach (216 patients) or secondary surgery (70 patients), according to the clinical spontaneous recovery at 4–6 months. Clinical improvement correlated with the increase of the compound muscle action potential amplitude (OR 3.76; CI 1.61–8.76), particularly in the S group (OR 7.25; CI 1.2–43.87), and with sensory nerve action potential amplitude in the NS group (OR 4.35; CI 1.14–16.69). No correlations were found with needle electromyography qualitative evaluations, changes in maximal voluntary recruitment, age, and gender.* Conclusions*. Nerve conduction studies (NCS) represent the more accurate neurophysiological markers of clinical outcome in patients with TN.* Significance*. Serial NCS assessments predict the functional recovery in TN, increasing the accuracy of peripheral nerves surgical decision-making process.

## 1. Introduction

Traumatic neuropathies (TN) due to work accidents, sports injuries, and high-speed road accidents are a common cause of disability and quality of life impairment in the young adult population [1, 2].

Peripheral nerve damage can be classified as neurotmesis (disruption of both axons and nerve sheath), axonotmesis (disruption of axons with preserved integrity of endoneurium, perineurium, and epineurium), or neuroapraxia (temporary damage of myelin sheath without damage of axons).

Open injuries with evidence of neurotmesis necessitate primary reconstructive surgery (S-I), while secondary surgical exploration (S-II) can be considered in closed lesions with poor clinical and electrodiagnostic (EDX) spontaneous recovery in order to determine the extent of TN and perform the necessary microsurgical reconstruction [3].

EDX studies may be useful to evaluate distal muscle reinnervation, topography, and severity of TN [4]. However, only few correlations have been currently reported with nerve regeneration processes and no consensus exists regarding the most appropriate measure of functional recovery [2, 5–8]. The identification of neurophysiological markers with clinical prognostic value would be thus of the utmost relevance in the clinical and surgical management of TN.

This large observational multicentre study reports the three years prospective data of 307 TN patients treated with surgery and/or conservative approaches in six Italian specialized centres (Italian Traumatic Neuropathy Network). Our main aims were to identify predictors of clinical functional recovery and to evaluate the clinical prognostic role of EDX studies in patients receiving medical treatment, primary and/or secondary surgery.

## 2. Methods

### 2.1. Subjects

Patients referred to the Italian Traumatic Neuropathy Network between January 2010 and December 2011 were enrolled in the study after signing a written informed consent (Ethical Committee Approval Prot. number 0023037, number CEI-227) and the evaluation of study inclusion criteria, namely, age ≥14 years-old, compliance with diagnostic tests, and no previous evidence of neuropathy.

### 2.2. Study Design

**Figure 1:**
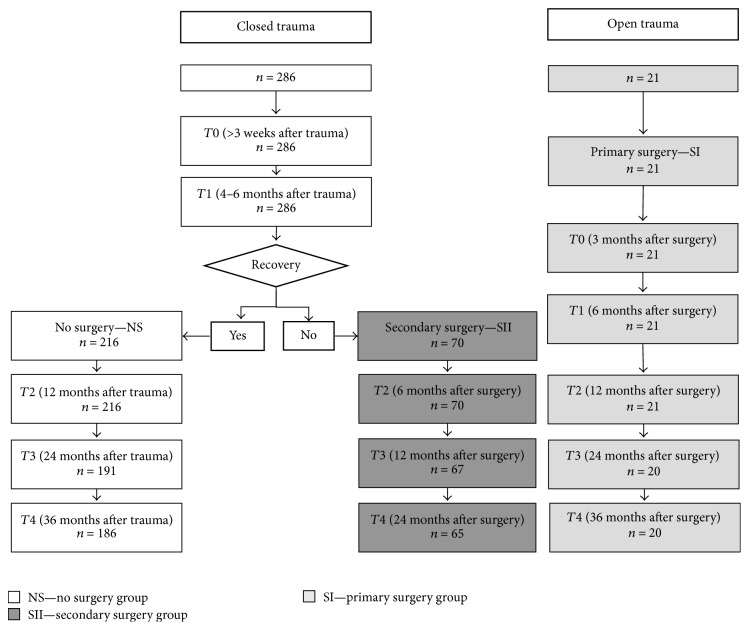
Study flowchart: clinical and neurophysiological timing.

Baseline (*T*0) clinical/EDX evaluations were performed after ≥3 months from surgery in the group of patients treated with S-I (open lesions with evidence of neurotmesis) and after ≥3 weeks from the TN event in the group of patients treated with nonsurgical (NS) approach or S-II (close lesions with poor spontaneous recovery after 4–6 months of follow-up). Patients received regular clinical/EDX assessments at *T*1 (4–6 months), *T*2 (12 months), *T*3 (24 months), and *T*4 (36 months), with the aim of evaluating signs of clinical and/or neurophysiological recovery (Figure 1).

### 2.3. Clinical/EDX Assessment

Patients were evaluated by means of the modified Rankin Scale (mRS) [9], at baseline and at different time-points (Figure 1), considering a decrease of ≥1 point in the mRS as a follow-up clinical improvement. EDX studies were performed in accordance with the protocols suggested by Ferrante and Wilbourn [10] for BP injuries and by Preston and Shapiro [11] for radiculopathy and mononeuropathies: needle electromyography (EMG) included observation of any abnormal spontaneous activity, qualitative evaluation of motor unit action potentials (MUAPs), and evaluation of maximal voluntary recruitment (MVR), which was rated as (a) “absent” (no MUAPs); (b) “discrete” (individual MUAPs); (c) “reduced” (decreased recruitment of MUAPs); or (d) “normal” (interference). Nerve conduction studies were carried out using surface electrodes to measure compound muscle action potential (CMAP) and sensory nerve action potential (SNAP) amplitudes. Findings different by more than two standard deviations (SD) from each laboratory normative data were considered abnormal.

Follow-up EDX improvement was defined as follows: (a) CMAP and SNAP amplitudes = increase of at least 16% versus baseline values [12]; (b) MVR pattern = increase of MVR in at least 1 target muscle; and (c) reinnervation MUAPs = evidence of ≥2 reinnervation MUAPs in at least 1 target muscle. Skin temperature was measured with a digital thermometer and kept constantly above 32°C with an infrared lamp.

### 2.4. Statistical Analysis

Mann-Whitney *U*, Wilcoxon rank sum, and Cramer's *V* tests were used for comparison between and within groups, while a multiple logistic regression model was used to calculate the prognostic accuracy of each EDX marker in the prediction of clinical recovery, considering mRS improvement as a dependent variable and EDX outcome measures as predictive (independent) variables. Associations were analyzed by crude evaluations and then specified (reevaluated), considering the most important confounding factors (i.e., sex, age, site of injury, dominant hemisphere, and polytrauma surgery). All *p* values reported are two-tailed, considering 0.05 as statistical threshold. Analyses were performed with SPSS Statistics 21.0 for Mac.

## 3. Results

### 3.1. Baseline Data

Baseline clinical and EDX data were available for 307 consecutive patients (Table 1) for a total of 444 TN (Table 2). Mechanisms of injury included contusion (36%), stretching (35%), transection (15%), ischemia (13%), and avulsion (1%), resulting in 117 plexopathies (72% axonotmesis; 26% neurotmesis; and 2% neuroapraxias), 75 root lesions (12% axonotmesis; 76% neurotmesis; and 12% neuroapraxias), and 252 nerve lesions (64% axonotmesis; 32% neurotmesis; and 4% neuroapraxias).

### 3.2. Follow-Up Data

Complete clinical and EDX follow-up data were available for 307 patients (Figure 1) at *T*0, *T*1, and *T*2; 29 patients dropped out at *T*3 and 36 patients dropped out at *T*4: 91/307 patients received surgery (21 S-I and 70 S-II) and 216/307 were treated with conservative medical approach (Table 1). Indications to surgery included (a) double-level BP + root lesions (82% of cases received surgery); (b) root avulsion (56% of cases received surgery); (c) double-level BP + nerve lesions (44% of cases received surgery); (d) BP lesions (29% of cases received surgery); (e) and nerve lesions (24% of cases received surgery).

According to the multiple regression analysis of clinical outcome (Table 3), the increase of CMAP amplitude correlated with mRS clinical improvement (OR 3.76; CI 1.61–8.76), especially in the S group (OR 7.25; CI 1.2–43.87) and in patients with BP lesions (OR 9.65; CI 1.64–56.75). Moreover, SNAP amplitude correlated with clinical improvement in the NS group (OR 4.35; CI 1.14–16.69).

No correlations were found between age, gender, or dominant hemisphere and clinical functional outcome, while surgical treatment per se was associated with worse clinical outcome (OR 0.27; CI 0.12–0.61), reflecting the more severe baseline clinical conditions (Table 1).

The improvement of at least one EDX marker was associated with a more accurate prediction of clinical recovery in NS versus S groups (*p* < 0.001): 64% of patients in the NS group versus 31% of patients in the S group reported a clinical and EDX improvement (*p* < 0.001), while 23% of patients in the NS group versus 53% in the S group reported only EDX improvement (*p* < 0.001), 3% of patients in the NS group versus 5% in the S group reported only clinical improvement (*p* = 0.3), and 10% of patients in the NS group versus 11% in the S group reported no clinical or EDX improvement (*p* = 0.8).

## 4. Discussion

This prospective multicentre study reports the 36-month follow-up data of 307 patients with TN, including plexopathies, root avulsions, and peripheral nerves lesions. The main objective was to evaluate the prognostic role of EDX on nerve regeneration processes and identify prognostic neurophysiological markers of clinical recovery after surgical or conservative treatments. We found a correlation between the increase of SNAP amplitude and peripheral nerve spontaneous recovery and between CMAP amplitude and clinical improvement in S-I and S-II groups. No significant correlations were found between reinnervation MUAPs or changes in the MVR pattern and clinical outcomes.

These data highlight the central role of nerve conduction studies in the assessment of the peripheral nerve regeneration processes [13], confirming the results of previous observational studies on traumatic radial nerve lesions and idiopathic/traumatic brachial plexopathies [14, 15]. Our data seem to suggest that the improvement of SNAPs might represent a precocious index of axonal regeneration, which could precede muscular reinnervation, strength recovery, and CMAP improvement. However, our findings showed a different profile of clinical/EDX functional recovery in patients treated with surgical or conservative treatments, with a higher prevalence of isolated EDX amelioration in the surgical groups. The more severe baseline condition of patients who received surgery, characterized by neurotmesis and/or severe axonotmesis, and frequently involving multiple nerves could partially account for these results. However, these data may also confirm the importance of an appropriate surgical timing, especially in proximal lesions involving cervical roots and BP, which should reinnervate distal target muscles before irreversible changes occur.

In conclusion, an individual clinical approach is required in TN patients, so that a correct diagnosis (level and site) is achieved, optimal therapy (conservative versus surgical) is planned and, in case of poor clinical recovery, the most appropriate timing for surgical procedures is decided. Great importance should be given to the first EDX evaluation, which might provide important information on the severity of damage, while serial EDX assessments might be helpful in following the peripheral nerve recovery; a gradual increase of SNAP amplitude may suggest a conservative treatment, while surgical exploration is recommended in patients with poor spontaneous recovery after 6 months.

Standardized clinical/EDX protocols seem advisable: (a) patients with open lesions treated with peripheral nerves primary surgery require an accurate monitoring of CMAP amplitude, which represents the most sensible indicator of clinical recovery; (b) patients with closed trauma should be carefully evaluated with clinical/EDX assessments, in order to identify the site and level of the nerve injury and monitor the CMAP and SNAP amplitudes. The lack of improvement after 4–6 months is a negative prognostic factor suggesting secondary surgical exploration.

## Figures and Tables

**Table 1:** TN patients: demographic features, aetiology, mechanism, and site of injury.

	All patients *n* = 307 (232 males/75 females)	NS group *n* = 216 (153 males/63 females)	S group *n* = 91 (79 males/12 females)
Dominant hemisphere	229 left/78 right	147 left/69 right	82 left/9 right

Aetiology			
Motorcycle accident	80	34	46
Car accident	55	46	9
Accident at work	47	39	8
Iatrogenic lesion	31	27	4
Domestic accident	24	15	9
Accidental fall	22	19	3
Bicycle accident	12	9	3
Sports injury	10	10	0
Suicide attempt	4	2	2
Burns	4	4	0
Others	18	11	7

Site of injury			
Brachial plexus—BP	87	62	25
Root	9	4	5
Nerve	182	139	43
Double-level BP + root	17	3	14
Double-level BP + nerve	9	5	4
Lumbar plexus	3	3	0

Polytrauma	213	158	55

TN: traumatic neuropathies; *n*: number of patients; NS: nonsurgical; S: surgical; and BP: brachial plexus.

**Table 2:** Sites of injury.

	All TN *n* = 444	NS group *n* = 276	S group *n* = 168
Brachial plexus	113	70	43

Lumbar plexus	4	4	0

Cervical root	75	18	57

Nerve	252	184	68
Radial	43	31	12
Peroneal	42	37	5
Ulnar	35	23	12
Median	34	18	16
Sciatic	19	18	1
Axillary	18	13	5
Musculocutaneous	11	6	5
Suprascapular	11	7	4
Tibial	9	7	2
Digital	9	6	3
Sural	5	4	1
Facial	4	4	0
Supraorbital	3	3	0
Long thoracic	3	2	1
Femoral	3	2	1

TN: traumatic neuropathies; *n*: number of lesions; NS: nonsurgical; and S: surgical.

**Table 3:** Multiple regression analysis of clinical outcome.

	All subjects OR (95% IC)	NS group OR (95% IC)	S group OR (95% IC)	BP lesion OR (95% IC)	Nerve lesion OR (95% IC)
Gender	1.29 (0.5–3.3)	2.02 (0.58–6.99)	0.48 (0.06–3.66)	1.42 (0.19–10.65)	1.12 (0.37–3.41)

Age	1.00 (0.98–1.03)	1.00 (0.98–1.04)	0.98 (0.94–1.03)	1.05 (0.99–1.11)	0.99 (0.96–1.02)

Dominant hemisphere	2.19 (0.57–8.47)	4.84 (0.50–47.01)	1.50 (0.17–12.07)	23.59 (0.77–720.51)	1.44 (0.32–6.5)

Surgery	**0.27** **(0.12–0.61)**	—	—	0.12^*∗*^ **(0.02–0.58)**	0.56 (0.2–1.6)

Polytrauma	**3.15** (1.27–7.83)	2.10 (0.60–7.37)	8.41^*∗*^ **(1.48–47.69)**	5.10 (0.44–59.63)	4.02^*∗*^ **(1.47–10.99)**

BP lesions	0.44 (0.06–3.14)	0.26 (0.03–2.18)	NA	—	0.54 (0.08–3.77)

Nerve lesions	1.69 (0.23–12.24)	0.43 (0.05–3.82)	NA	3.12 (0.24–40.05)	—

SNAP amp. increase	2.28 (0.96–5.43)	4.35^*∗*^ **(1.14–16.69)**	1.98 (0.43–9.06)	2.81 (0.62–12.77)	1.35 (0.43–4.27)

CMAP amp. increase	3.76^*∗*^ **(1.61–8.76)**	2.67 (0.91–7.85)	7.25^*∗*^ **(1.2–43.87)**	9.65^*∗*^ **(1.64–56.75)**	2.78 (0.94–8.17)

MVR improvement	0.79 (0.31–2.02)	1.14 (0.31–4.24)	0.43 (0.07–2.54)	4.92 (0.47–51.70)	0.41 (0.10–1.61)

Reinnervation MUAPs	2.04 (0.60–6.92)	3.33 (0.75–14.84)	1.01 (0.07–15.92)	7.92 (0.29–215.10)	2.15 (0.55–8.43)

Multiple regression analysis of clinical outcome measure (modified Rankin scale) improvement, as dependent variable. Predictive (independent) variables include gender, age, dominant hemisphere, surgery, polytrauma, BP (brachial plexus) lesions, nerve lesions, SNAP (sensory nerve action potential) amplitude increase, CMAP (compound muscle action potential) amplitude increase, MVR (maximal voluntary recruitment) improvement, and reinnervation MUAPs (motor unit action potentials).

NS: nonsurgical; S: surgical; OR: odds ratio; CI: confidence interval; NA: information not sufficient for reliable estimates; and amp.: amplitude. ^**∗**^Statistical significance (*p* < 0.05).
